# Imputation and quality control steps for combining multiple genome-wide datasets

**DOI:** 10.3389/fgene.2014.00370

**Published:** 2014-12-11

**Authors:** Shefali S. Verma, Mariza de Andrade, Gerard Tromp, Helena Kuivaniemi, Elizabeth Pugh, Bahram Namjou-Khales, Shubhabrata Mukherjee, Gail P. Jarvik, Leah C. Kottyan, Amber Burt, Yuki Bradford, Gretta D. Armstrong, Kimberly Derr, Dana C. Crawford, Jonathan L. Haines, Rongling Li, David Crosslin, Marylyn D. Ritchie

**Affiliations:** ^1^Department of Biochemistry and Molecular Biology, Center for Systems Genomics, The Pennsylvania State UniversityPennsylvania, PA, USA; ^2^Division of Biomedical Statistics and Informatics, Department of Health Sciences Research, Mayo ClinicRochester, MN, USA; ^3^The Sigfried and Janet Weis Center for Research, Geisinger Health SystemDanville, PA, USA; ^4^Center for Inherited Disease Research, John Hopkins UniversityBaltimore, MD, USA; ^5^Cincinnati Children's Hospital Medical CenterCincinnati, OH, USA; ^6^Department of Medicine, University of WashingtonSeattle, WA, USA; ^7^Center for Human Genetics Research, Vanderbilt UniversityNashville, TN, USA; ^8^Department of Epidemiology and Biostatistics, Case Western UniversityCleveland, OH, USA; ^9^Division of Genomic Medicine, National Human Genome Research InstituteBethesda, MD, USA

**Keywords:** imputation, genome-wide association, eMERGE, electronic health records

## Abstract

The electronic MEdical Records and GEnomics (eMERGE) network brings together DNA biobanks linked to electronic health records (EHRs) from multiple institutions. Approximately 51,000 DNA samples from distinct individuals have been genotyped using genome-wide SNP arrays across the nine sites of the network. The eMERGE Coordinating Center and the Genomics Workgroup developed a pipeline to impute and merge genomic data across the different SNP arrays to maximize sample size and power to detect associations with a variety of clinical endpoints. The 1000 Genomes cosmopolitan reference panel was used for imputation. Imputation results were evaluated using the following metrics: accuracy of imputation, allelic *R*^2^ (estimated correlation between the imputed and true genotypes), and the relationship between allelic *R*^2^ and minor allele frequency. Computation time and memory resources required by two different software packages (BEAGLE and IMPUTE2) were also evaluated. A number of challenges were encountered due to the complexity of using two different imputation software packages, multiple ancestral populations, and many different genotyping platforms. We present lessons learned and describe the pipeline implemented here to impute and merge genomic data sets. The eMERGE imputed dataset will serve as a valuable resource for discovery, leveraging the clinical data that can be mined from the EHR.

## Introduction

Imputation methods are widely used for inferring unobserved genotypes in a genotypic dataset using haplotypes from a more densely genotyped reference dataset (Browning, 2008; Howie et al., 2009, 2011, 2012; Li et al., 2009). This process is particularly important when combining or performing meta-analysis on data generated using multiple different genotyping platforms. Imputation allows for the utilization of a reference dataset and a genotyping backbone, identifying what the unobserved likely SNPs are using patterns of linkage disequilibrium (LD) amongst surrounding markers. Multiple imputation software packages and algorithms have been developed for imputing SNPs (Browning, 2008; Browning and Browning, 2009; Li et al., 2010; Delaneau et al., 2013) (Howie et al., 2012, 2009). Although each method has clear strengths and limitations, a single “best-practice” imputation software package has not yet emerged as each tool will have different assumptions, benefits and weaknesses.

In the electronic MEdical Records and GEnomics (eMERGE) network (Gottesman et al., 2013) funded by the National Human Genome Research Institute (NHGRI), multiple genotyping platforms were have been used to generate genome-wide genotype data for thousands of patient samples and a variety of phenotypes extracted from electronic health records (EHR). To allow for either meta-analysis across the eMERGE sites or a combined mega-analysis whereby all of the eMERGE datasets are combined in a single analysis, imputation is essential to fill in the missing genotypes caused by using disparate genotyping platforms. The eMERGE Coordinating Center (CC) at the Pennsylvania State University performed genotype imputations for the eMERGE Phase-II project data [which includes all samples from eMERGE-I (McCarty et al., 2011; Zuvich et al., 2011), and eMERGE-II (Gottesman et al., 2013; Overby et al., 2013) using two different imputation pipelines: (1) BEAGLE (Browning and Browning, 2009) version 3.3.1 for phasing and imputation, and (2) SHAPEIT2 (version r2.644) (Delaneau et al., 2013) for phasing in combination with IMPUTE2 (version 2.3.0) software (Howie et al., 2012) for imputation. Imputation was performed for all autosomes, with a cosmopolitan reference panel selected from the 1000 Genomes Project (1000 Genomes Project Consortium et al., 2012). BEAGLE used the October 2011 release and IMPUTE2 used the March 2012 release based on the timing of when imputations were performed. We did not perform X-chromosome imputations as part of this paper but the imputation of the X chromosome for these datasets is currently in progress. In these imputations, 1000 Genomes cosmopolitan reference panel was selected whereby 1092 samples from multiple race, ethnicity and ancestry groups were included in the reference panel. Using a cosmopolitan reference panel is advantageous when imputing data based on multiple ancestry or mixed-ancestry groups (Howie et al., 2011), as is the case in eMERGE datasets. To maximize our use of computational resources and allow for high quality imputations, the CC imputed the data as they were submitted to the CC, in datasets by site and genotyping platform, using the cosmopolitan panel from the 1000 Genomes.

Imputed data from all eMERGE sites were merged based on the set of intersecting SNPs present in all datasets. For the merging process, datasets that were not genotyped on dense, genome wide platform, and the datasets with fewer than 100 samples were not included as these sets routinely showed much lower quality imputation results (See Materials and Methods; additional data not shown). For example, genotyping panels containing markers in only some regions of the genome [such as the Illumina MetaboChip (Voight et al., 2012)] do not provide a suitable backbone for high quality genome-wide imputation. We looked at the quality of imputation in each of these datasets by the estimated imputation “info” score (See Results). Additionally, for datasets with very small sample size and/or not genotyped densely, median info score was close to 0 (For e.g., CHOP Illumina OmniExpress dataset with only 32 samples had median info score of 0.007), so we excluded these datasets from the merged data. After imputation and merging of the datasets, quality control procedures were implemented to create high quality, analysis-ready data set for genome-wide association studies.

Here we describe the imputation pipelines implemented using BEAGLE and SHAPEIT2/IMPUTE2; provide results of the two imputation pipelines; and describe the quality control procedures after merging multiple imputed datasets. Numerous lessons were learned along the way for each of these imputation pipelines and we share all of the challenges encountered in the project. The imputation and quality control procedures resulted in unique and comprehensive a dataset of over 50,000 samples with genotypes imputed to the 1000 Genomes reference panel, all linked to de-identified EHR to allow for a vast array of genotype-phenotype association studies.

## Materials and methods

### Study data

The eMERGE network consists of seven adult sites and two pediatric sites, each with DNA databanks linked to EHR. Each site in the network has a set of at least 3000 samples that have been genotyped on one or more genotyping platforms (Gottesman et al., 2013). Table 1 provides a summary of the number of samples from each site and the genotyping platforms used. Previous studies have shown that the quality of input genotype data does not affect imputation quality in a significant manner (Southam et al., 2011), but nevertheless we selected the genomic data sets for the current imputation study that had all undergone the pre-processing recommended by the eMERGE CC to eliminate samples and SNPs with call rates less than 99–95% depending on the coverage of genotyping for each platform (Zuvich et al., 2011). Minor allele frequency (MAF) threshold of 5% was also applied. This ensures that only high quality data were considered for imputation and downstream analyses.

**Table 1:** **Sample summary across all eMERGE datasets**.

**Sample set**	**Genotyping platform**	**Samples for imputations**	**Samples in merged data set**
**ADULT DNA SAMPLES**			
eMERGE-I 1M	Illumina 1M	2634	2634
eMERGE-I 660	Illumina 660	18663	16029
Geisinger OMNI	Illumina HumanOmni Express	3111	3111
Geisinger Metabochip	Metabochip	918	0
Mayo Clinic	Illumina Human 610, 550, and 660W Quad-v1	3149	3118
Mt. Sinai AA	Affymetrix 6.0	863	863
Mt. Sinai EA	Affymetrix 6.0	700	700
Mt. Sinai HA	Affymetrix 6.0	1212	1212
Mt. Sinai OMNI_AA	Illumina HumanOmni Express	3515	3515
Northwestern University	Illumina HumanOmni Express 12v1_C	3030	2951
Vanderbilt University	Illumina HumanOmni Express 12v1_C	3565	3461
Group Health/ACT	Illumina HumanOmni Express	398	398
Group Health/NWIGM	Illumina 660W-Quad Beadchip	341	333
Marshfield Clinic	Affymetrix/Illumina 660	500	500
Total for adult DNA samples		42,599	38,824
**PEDIATRIC DNA SAMPLES**			
CCHMC	610/660W/AffyA6/OMNI1/OMNI5	5558	4322
BCH	Affymetrix Axiom	1038	1038
CHOP	550/610/Beadchip/AffyA6/AffyAxiom/OmniExpress	7695	6850
Total for pediatric DNA samples		14,291	12,210
Total		56,890	51,035

“Samples for imputations” column contain number of samples that were obtained by coordinating center at different time points. “Samples in merged dataset” contain number of unique samples that were used in merged dataset. For the samples that were genotyped on multiple platforms, sample on platform with high genotype efficiency was used in merged dataset.

Several eMERGE sites genotyped duplicate samples on multiple different genotyping platforms, for quality control purposes. A total of 56,890 samples were submitted to the eMERGE CC for imputation, out of which 53,200 samples were unique. All of these samples were genotyped and deposited to CC at different times, so imputation was performed as the datasets arrived. This resulted in imputing some datasets with fewer than 100 samples. When the dataset was less than 100 samples, we included the 1000 Genomes dataset with the study data during phasing. We imputed all samples; however, for the purpose of merging the data, we only merged high quality datasets (defined by having masked analysis concordance rate greater than 80%; described in more detail below). We included only one sample from pairs of duplicates; specifically the sample genotyped on the higher density genotyping platform. Our final merged dataset contains 51,035 samples. Samples that had low quality due to either of the following two reasons were not included:

1. Samples not genotyped on dense, genome-wide genotyping platform (e.g., the MetaboChip).
2. Sample size of the dataset on the specific platform for phasing was fewer than 100 (as recommended in SHAPEIT2 guidelines).

A small number of samples were also genotyped for two SNPs (rs1799945 and rs1800562) using commercially available 5′-nuclease assays (TaqMan® Assay; Life Technologies). Genotyping reactions were carried out in 10 μl volumes in an ABI 7500 Fast Real-Time PCR System (Life Technologies). The genotypes were called using ABI 7500 software version 2.0.4 (Life Technologies). These data were used to evaluate the concordance of imputed genotypes with TaqMan generated genotypes.

### Pre-imputation data processing

The quality of imputation relies on the quality of the reference panel as well as the quality of the study data. To ensure high data quality, there are a number of steps that were taken before imputation begins. At the start of the BEAGLE imputation, the GENEVA HAPO European Ancestry Project Imputation Report (Geneva_Guidelines^1^) by Sarah Nelson through GENEVA (Gene-Environment Association Studies) was used as a guide and a starting point for implementation of the eMERGE imputation pipeline. GENEVA is an NIH-funded consortium of sixteen genome-wide association studies (GWAS) and this guide served as the basis to begin the eMERGE Phase-II imputation process.

### Converting reference panel and study data to the same genome build

The genotype data were initially accessed from binary PLINK files (Purcell et al., 2007). All SNP names and locations for the genotypic data being imputed had to be specified based on the same genome build, as well as the same genome build of the reference genome. The Genome Reference Consortium Human build 37 (GRCh37 or build 37) is the reference genome used in our study (2010). Some eMERGE sites had their data in build 37, while others were still in build 36. Any datasets that were not in build 37 were first converted from build 36 to build 37 using the Batch Coordinate Conversion program liftOver (Karolchik et al., 2011) via the following steps:

1. SNPs with indeterminate mappings were removed (either unknown chromosome and/or unknown position) in build 37.
2. SNP names were updated.
3. The chromosome positions were updated.
4. The base pair positions were updated.

The program liftOver is a tool developed by the Genome Bioinformatics team at the University of California, Santa Cruz (UCSC) to convert genome coordinates and genome annotation files between assemblies. This process ensures that all study data from eMERGE sites and the 1000 Genomes reference data are referring to SNPs by the same alleles and genome location.

### Checking strand

Study and reference data allele calls must be on the same strand for proper imputation, however the strand could vary from study site to study site due to genotyping platform and calling algorithm. High quality imputation is exceptionally reliant upon the study and reference data allele calls to be on the same physical strand of DNA in respect to the human genome reference sequences (“reference”). Datasets could have different notations depending on the genotyping platform and the calling algorithm. For example, Genome Studio will allow the user to create genotype files using different orientations. In addition, some users may use custom genotype callers—not provided by the genotyping chip manufacturer. For example, some platforms use the forward strand of the human genome assembly and some use Illumina's TOP alleles, and some use Illumina's AB alleles (Illumina TechNote^2^). To identify the SNPs requiring a strand flip to convert the forward allele to the “+” strand of the human genome reference assembly so that all sites were consistent in terms of the same strand, we used the BEAGLE strand check utility for BEAGLE imputations and the SHAPEIT2 strand check for IMPUTE2 imputations even though IMPUTE2 automatically addresses ambiguous strand alignments by comparing allele labels. During strand check, alleles are changed to their complementary alleles (C-G and A-T) based on three criteria: (a) the observed alleles, (b) minor allele frequencies (MAF), and (c) linkage-disequilibrium (LD) pattern within 100-SNP windows. SNPs where MAF and LD patterns are inconsistent and also cannot be resolved by flipping, those SNPs are discarded from the dataset. Before phasing, we subset the data by chromosomes and also flipped strand for the SNPs to align the dataset with “+” strand so that it corresponds to reference panel strand correctly.

### Phasing

Haplotype phasing is the next step after ensuring that all data was using the same strand, identifying alleles co-localized on the same chromosome. BEAGLE performs phasing jointly with imputations. “Pre-phasing” indicates that a computational step is implemented prior to imputation where haplotype phase is estimated for all of the alleles. We utilized a pre-phasing approach because it helps to make the process of imputation faster, and the phased data can be used for any future imputation of the data. Improved reference panels will be introduced over time, and thus having the data saved pre-phased for imputation can speed up later imputation of the data. Phasing the data can introduce some error to the imputations, because of any haplotype uncertainty (Howie et al., 2012).

For IMPUTE2 imputations, following “best practices” guidelines in the IMPUTE2 documentation (Howie et al., 2009) (Impute2, 2.3.0) we first phased the study data with the SHAPEIT2 haplotype estimation tool (Howie et al., 2012). We were able to reduce general runtime through using multiple computational processing cores via the “—thread” argument. A general example of the command line syntax used to run the SHAPEIT2 program on chromosome 22 using the “—thread” argument is shown below:


shapeit2 --input-ped StudyData_chr22.ped
StudyData_chr22.map \
--input-map genetic_map_chr22_combined_
b37.txt \
--output-max StudyData_chr22.haps
StudyData_chr22.sample \
--thread 2 --output-log shapeit_chr22.log


### Imputation using beagle

**Figure 1:**
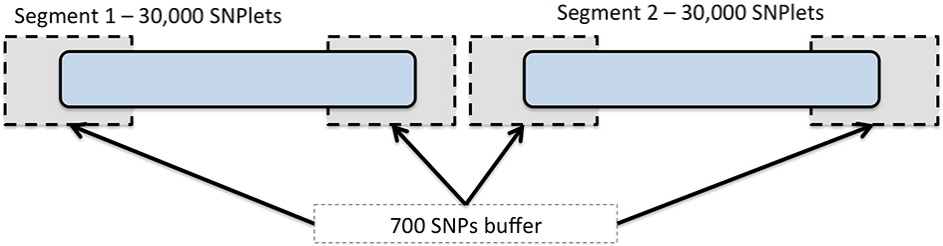
**Chromosome segmentation strategy for genome-wide imputation with BEAGLE**. Each chromosome was divided into SNPlets which included 30,000 SNPs with a buffer of 700 SNPs at each end.

To expedite the imputation using BEAGLE, we divided each chromosome into segments including 30,000 SNPs each (referred to as SNPlets), following one of several recommendations in the BEAGLE documentation (Browning and Browning, 2009) for imputing large data sets. A buffer region of 700 SNPs was added to each end of every SNPlet to account for the degradation in imputation quality that may occur at the ends of imputed segments. An illustration of this segmentation is shown in Figure 1. Partitioning was implemented by dividing the “.markers” files created at the end of the strand check into 1 “.markers” file for each SNPlet of 30,000 SNPs and a 700 SNP buffer region on either end. In each results file, the data for all SNPs in the buffer regions were removed such that each imputed SNP had results from only one segment. The SNP annotation and quality metrics file accompanying these data indicate to which segment each SNP was assigned.

Below is an example of the command line syntax used to run BEAGLE on the first segment of chromosome 22. The “phased=” argument corresponds to the 1000 Genome Project reference panel input file; the “excludemarkers=” argument points to a combined list of SNPs that are either (1) triallelic SNPs or (2) have reference MAF < 0.005. The “unphased=” argument points to a BEAGLE-formatted input file:


java -Djava.io.tmpdir=/scratch/tmp
-Xmx4700m -jar BEAGLE.jar \
unphased= chr22_mod.bgl \
phased= chr22.filt_mod.bgl \
markers= chr22_*.markers \
excludemarkers= allchr22_snpsexclude.txt \
lowmem=true verbose=true missing=0
out=out_chr22set1


### Imputation using IMPUTE2

**Figure 2:**
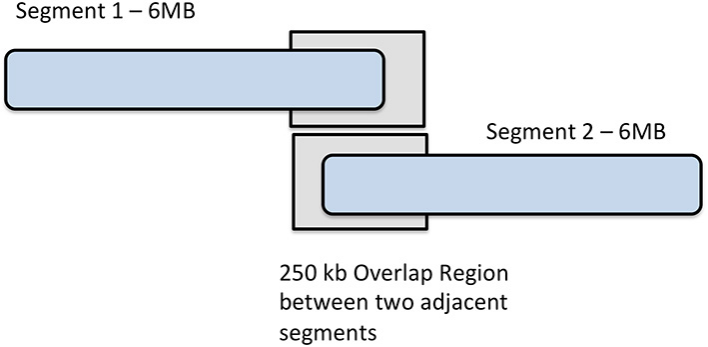
**Chromosome segmentation strategy for imputation with IMPUTE2**. Each chromosome was divided into 6 MB segments with 250 kbp overlap between them.

To perform imputation with IMPUTE2 on our phased data, we divided each chromosome into base pair regions of approximately 6 Mb in size, beginning at the first imputation target, as displayed in Figure 2. As a result, we partitioned 22 autosomes into 441 segments, ranging from only 7 segments on chromosome 21, to the largest number of segments (39) on chromosome 2. It is of interest to note that there were 36 segments on chromosome 1. It was beneficial to use this process of breaking the genotypic data into smaller regions because IMPUTE2 has been reported to have improved accuracy over smaller genomic regions and also separating data into segments helps allows for the parallelization of jobs over a multi-core compute cluster. Segments either overlapping the centromere or at the terminal ends of chromosomes were merged into the segment immediately upstream.

IMPUTE2 labels SNPs by the panels in which they have been genotyped. Each label denotes a specific functional role. SNPs that have genotype data only in the reference panel are labeled *Type 0* or *Type 1* (for phased and unphased reference panels, respectively), whereas SNPs that have genotypes in the study dataset are labeled *Type 2*. These are considered SNPs for the imputation basis. *Type 2* SNPs dictate which reference panel haplotypes should be “copied” for each individual; then, the reference panel alleles at *Type 0/1* SNPs are used to fill in the missing genotypes of the individual.

As recommended by the IMPUTE2 guidelines, we ensured that each base pair region that was imputed contained at least some observed (type-2) SNPs. To utilize type-2 SNPs for estimating haplotype structure, a buffer region on both sides of segments is required. 250 kb buffer region is default for IMPUTE2 so we used the default buffer size of 250 kb for eMERGE imputations. By default, IMPUTE2 flanks imputation segments with a 250 kb buffer, where type-2 SNPs are used to estimate haplotype structure. We used the default buffer size of 250 kb for imputations.

An example of the command line syntax we used to run first 6 MB segment (pre-phased) for chromosome 22 by IMPUTE2 (version 2) is shown below:


impute2 -use_prephased_g -m genetic_map_
chr22_combined_b37.txt \
-h ALL_1000G_phase1interim_jun2011_chr22_
impute.hap.gz \
-l ALL_1000G_phase1interim_jun2011_chr22_
impute.legend.gz \
-int 16000001 2.1e+07 -buffer 500 -allow_
large_regions \
-known_haps_g StudyData_chr22.haps \
-filt_rules_l Study_data.maf<0.001
-align_by_maf_g \
-o StudyData_chr22.set1.gprobs \
-i StudyData_chr22.set1.metrics -verbose


## Results

### Computation time and memory usage

**Figure 3:**
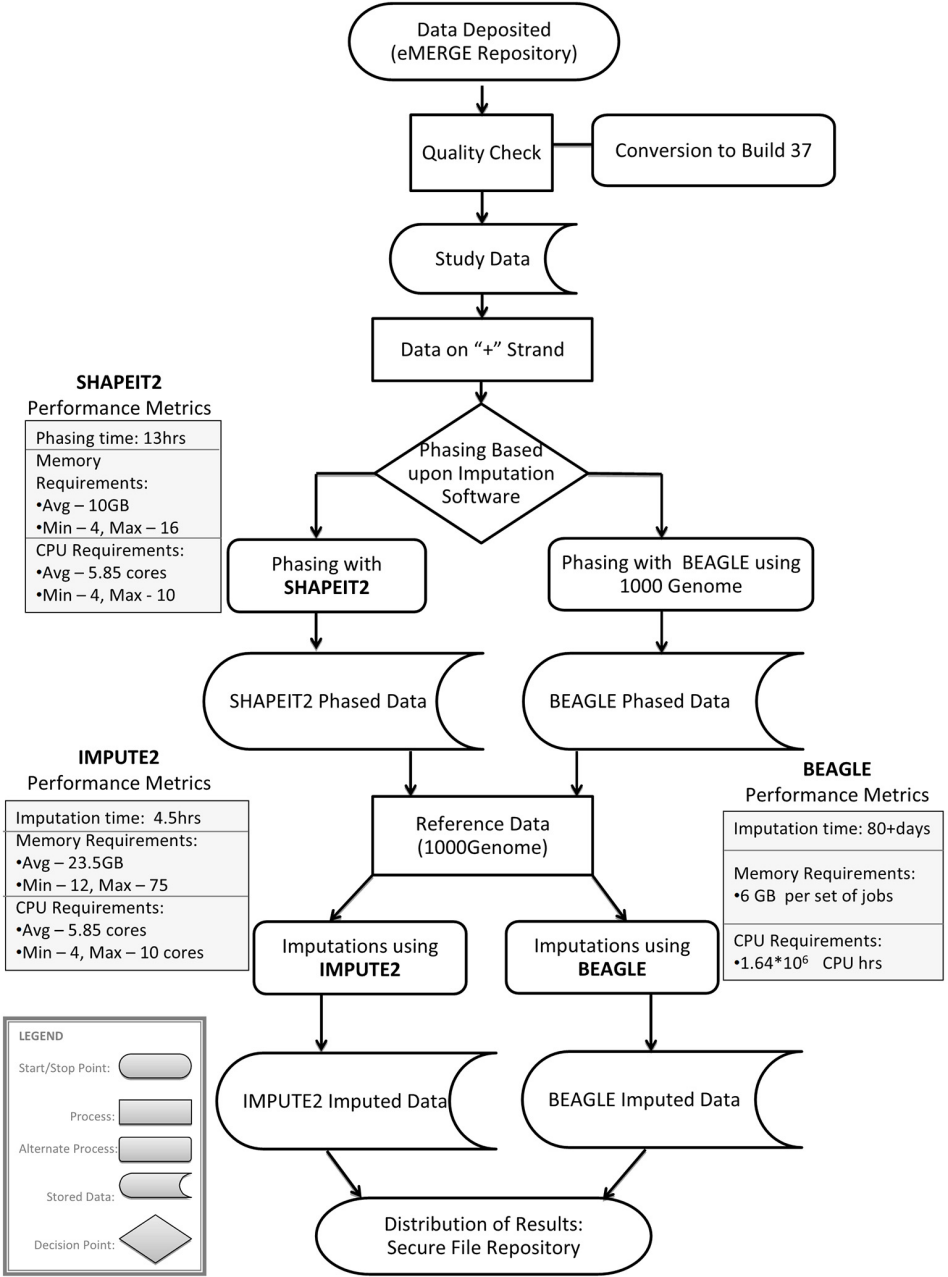
**Workflow and performance metrics for imputation with BEAGLE and IMPUTE2**.

Imputation jobs were run in parallel across several high-performance computing clusters; specialized systems were chosen depending on the memory and processor requirements of the software and the size of the datasets. Figure 3 shows the workflow of both imputation methods using the different software and how the performance results and computational requirements differed for each. Each job required between 4 and 24 GB RAM and from 4 to 80 CPUs (cores). The number of jobs submitted to be run in parallel also ranged from a few 100 to several 1000 at a time according to the sample size of each data set. Table 2A provides information on one of the computing clusters that were used to perform these extensive imputations by the eMERGE CC. Table 2B lists maximum time and memory from each of the datasets that was required to run both imputation and phasing. One thing to note here is that according to available sources at the time of running specific job, different CPU cores were utilized.

**Table 2:** **Computational resources used for conducting the imputations**.

**(A) Penn State Lion XG: systems specifications**					
**Component**	**Server**	**Quantity**	**Processor**	**Number of processor cores**	**Memory (GB)**
Login Node	Dell PowerEdge R620	1	Intel Xeon E5-2670 2.6 GHz	16	64
Compute Node	Dell M620	48	Intel Xeon E5-2665 2.4 GHz	16	128
Compute Node	Dell M620	48	Intel Xeon E5-2665 2.4 GHz	16	64
Compute Node	HP BL460c Gen8	96	Intel Xeon E5-2665 2.4 GHz	16	64
**(B) Phasing and imputation time and RAM required for each dataset**					
**Site_name**	**#Samples**	**Phasing time (maximum seconds)**	**Phasing RAM**	**Imputation_time (maximum seconds)**	**Imputation RAM**
eMerge-I-1M	2634	199984	4	7728	20
eMerge-I-660	18663	17263	4	35517	75
BCH	1038	22963	4	2380	12
Geisinger_Metabochip	918	55002	8	6011	24
Geisinger_OMNI	3111	2010	8	7397	20
Grouphealth_ACT	398	62141	8	1330	16
Grouphealth_NWIGM	341	8063	6	730	20
Mayo	6307	5627	6	21172	16
MtSinai_EA	700	130737	16	3617	30
MtSinai_AA	863	33832	10	2308	12
MtSinai_HA	1212	50884	10	3120	12
MtSinai_OMNI	3515	52000	16	13276	12
NU	3030	311211	32	6089	24
Vanderbilt	3565	19392	12	10583	20
Total CCHMC	4322	82450	12	4310	28
Total CHOP	6850	74501	12	7768	30

The largest variance for computing resource requirements was in the computational time required on the same cluster computing systems for the two different pipelines. Previous studies have compared both BEAGLE and IMPUTE2 programs based on the quality and imputation times (Pei et al., 2008; Howie et al., 2009, 2011; Nothnagel et al., 2009). Our work similarly showed that IMPUTE2 ran much faster than BEAGLE. For BEAGLE imputations, SNPlet runtimes varied between 40 and 200 h, on average using 6 GB of memory for each job for a total of 1.64 × 10^6^ CPU hours.

In summary SHAPEIT2 and IMPUTE2 processing, took only 13 h on an average for phasing using 10 GB memory with a maximum of 16 CPUs (4 cluster computing nodes where each node had 4 CPUs). Similarly imputations on average could be completed in 4.5 h of time using 24 CPUs (across multiple cluster nodes). For processing the final merged set, approximately 80 CPUs were required. The total computational time required for the SHAPEIT2 and IMPUTE2 processing was less than 600 CPU hours. Using the pre-phasing approach, imputation time was decreased by more than 10–fold with the unfortunate side-effect of utilizing intensive memory.

### Comparision of beagle and IMPUTE2

BEAGLE and IMPUTE2 methods have been compared extensively by previous studies of a single ancestry (i.e., European or African) and using a cosmopolitan reference panel (Browning and Browning, 2009; Howie et al., 2009; Nothnagel et al., 2009; Jostins et al., 2011; 1000 Genomes Project Consortium et al., 2012). Initially, we planned to perform a direct comparison of the two imputation programs. We found that the resource requirements to do that were prohibitive, since the 1000 Genomes reference was updated in between our BEAGLE runs and our IMPUTE2 runs. This update presented a conundrum since the update includes a large number of InDels and our proposed downstream analyses would be improved by using the updated reference set. Due to BEAGLE's compute intensive implementation we did not have the compute resources or the time to repeat the imputation with the new reference dataset. Similarly repeating the IMPUTE2 runs using the old reference, even though it was much faster than BEAGLE was prohibitive in terms of compute time and storage space with a dataset of 55,000 samples. Therefore, we will provide only anecdotal differences that we observed between IMPUTE2 and BEAGLE. For more complete, direct comparisons of the two approaches, we direct the reader to some of the earlier studies mentioned above.

In our study dataset, we observed that IMPUTE2 is substantially faster than BEAGLE but they both achieved comparatively equal accuracy with a large reference panel, such as the 1000 Genomes. Our BEAGLE imputations were only performed for adult data, so to look at the frequency of high quality markers, we compare the counts to adult only data in IMPUTE2. We observed that 8,899,961 SNPs passed allelic *R*^2^ filter of 0.7 in BEAGLE imputations whereas for same data using IMPUTE2 imputations, 12,504,941 SNPs passed info score filter of 0.7. Lastly, we also observed that in BEAGLE imputed data at MAF = 0.05, there were SNPs with Allelic *R*^2^ value less than 0.6 whereas with IMPUTE2 imputed data all SNPs with MAF = 0.05, were above info score value greater than 0.6.

Keeping the huge computational advantage of IMPUTE2 as well as quality of imputation in mind, especially when dealing with the imputation of over 50,000 samples, we used IMPUTE2 for further imputations and analyses. Thus, in the remainder of the paper, we will describe the output and quality metrics that we observed for IMPUTE2 in the eMERGE dataset.

### Masked analysis

One of the greatest challenges with imputation is knowing how well it is working. A common strategy used to evaluate this is called “masked analysis.” In a masked analysis, a subset of SNPs that were actually genotyped in the study sample are removed, those SNPs are then imputed as though they were not genotyped, and then the imputed SNPs are compared to their original genotypes. The results of the imputation are contrasted with the original genotypic data, showing the degree of concordance between the original genotypic data and the imputed data after masking. This gives a good sense of how accurate the imputations are with respect to that set of SNPs. An additional way the results of masking and imputation are evaluated is to compare the allelic dosage of the original genotypic data with that of the allelic dosage in the imputed data. If there are three genotypes AA, AB, and BB, the allelic dosage for each individual can be represented as probabilities (P) of each of three genotypes via 2*P(AA) + 1*P(AB) + 0*P(BB) to obtain the expected allelic dosage from the original genotypic data and the observed allelic dosage for the masked and imputed genotype for each SNP. The correlation between the expected allelic dosages and the observed allelic dosages over all individuals can then be calculated at each masked SNP. This correlation metric is an exact variant of the imputation *R*^2^ metrics of MACH (Li et al., 2010) and BEAGLE, which corresponded with the IMPUTE “info” score which is calculated automatically as part of IMPUTE2. Here Type 2 SNPs are removed from imputation, and then imputed, and contrasted with imputation input. Thus, metric files from IMPUTE2 provide information from these masked SNP tests, including concordance and correlation metrics, and an “info” metric for having treated a Type2 the SNP as Type 0.

Overall concordance is vigorously impacted by the MAF and we say so on the grounds that for SNPs with MAF < 5% by simply allocating imputed genotypes to the major homozygous state would result in >90% concordance. Thus, there is an inclination of high concordance values at low MAF SNPs, where major homozygotes are prone to be imputed “correctly” just by chance. We observed approximately 99% average concordance in masked SNPs grouped by MAF.

### Orthogonal genotyping analysis

As another imputation quality check, we compared the genotypes generated in the imputation with those genotyped on orthogonal genotyping platforms. Two SNPs, rs1800562 and rs1799945 were genotyped using TaqMan by the genotyping facility at Geisinger Health System. The concordance between the TaqMan genotype and the imputed data was 98.9 and 98.3% for rs1800562 and rs1799945, respectively. These are very similar to results observed in the Marshfield Clinic PMRP where an orthogonal platform was used (Verma et al., 2014).

### Merging of imputed datasets

Prior to imputation, we explored the option of combining the raw genotype data based on overlapping SNPs from the multiple GWAS platforms. Unfortunately the number of overlapping SNPs was minimal (only 37,978 SNPs). This was not sufficient for imputation. Thus, we imputed each dataset based on site and platform individually. After imputing each study dataset, we attempted to merge all of the imputed datasets together to generate a mega-analysis ready dataset (combining all eMERGE sites together). The imputed data from all eMERGE sites studying adult-onset diseases were merged into one dataset and all pediatric data were merged into a second set. Future directions include combining adult and pediatric data. Imputed datasets were merged based on the set of intersecting markers [only markers that were of high quality in all of the imputed data were combined (i.e., info score >0.7)]. Duplicate samples were removed, whereby the highest quality version of the sample was maintained. For example, if a sample was genotyped on two platforms with different call rates, we kept the result from the platform with the higher call rate. Additionally, the low quality data were omitted from the final version of the merged data. Low quality of imputation was determined by assessing the masked concordance rates calculated from IMPUTE2. Notably, most data that were not genotyped on a dense, genome-wide platform (such as MetaboChip or Illumina HumanHap 550 Duo BeadChip) had masked concordance rates <80% (Nelson et al., 2013). The lower concordance was probably due to a lack of a uniform backbone or imputation basis to use for construction of the LD patterns for imputation. As such, those datasets were not included in merged dataset. Finally, as recommended in both the SHAPEIT2 and IMPUTE2 guidelines (Impute2^3^), small sample size datasets (<100 samples) did not achieve high quality imputations; thus, we excluded them from the merged data.

To merge all of the datasets together, we implemented a script that takes IMPUTE2-formatted input files and cross-matches them based on SNP position and alleles, rather than the marker label (as sometimes marker labels are not shared). For each matching position, allele1, and allele2, which are found in all inputs, the output is given the most common label from among the inputs. The script detects cases where there are different SNP labels for the same Chr:Pos and alleles and resolves it by treating these as equivalent markers which will be joined into one output line, using the marker label which has larger rs#. For cases where there are more than one position for the same SNP label, the script will then drop both of them.

Imputation results have multiple columns of information. The first five columns relate to Chromosome, SNP ID, base pair location, and the two SNP alleles, where the first allele indicated is assigned “allele A,” and the second is assigned “allele B.” The following three columns represent the genotype probabilities of the three-genotype classes (AA, AB, and BB) for each individual sample; a simulated example shown here:

Imputed genotype files contain three types of IMPUTE2 SNPs: Type 0 (imputation target); Type 2 (imputation basis); and Type 3 (study only). Accompanying information metrics files provide information on what type of SNP each SNP within the dataset was. Note there are no sample identifiers in the probabilities files, consequently it is important to utilize sample information documents provided to adjust imputed probabilities to sample data. Merged “info” or quality metrics file contains following information:

1. “snp_id” is always “---”which is how it often appears in the input files, and “rs_id” and “position” match the genotype output file.
2. “type” is the numeric minimum of the observed input values.
3. The other columns are all simple (equally weighted) averages of the input values, except that any −1 inputs are ignored (for example. the average of 0.5, −1, 0.3 is 0.4, ignoring the −1).
4. There is also a special case for the “exp_freq_a1” column for inputs which have alleles reversed compared to the first input (allele1 is not always major or minor allele); in that case the value is subtracted from 1.0 before going into the average so that we always report frequencies for minor allele in merged dataset.

### Imputed data statistics for IMPUTE2

**Figure 4:**
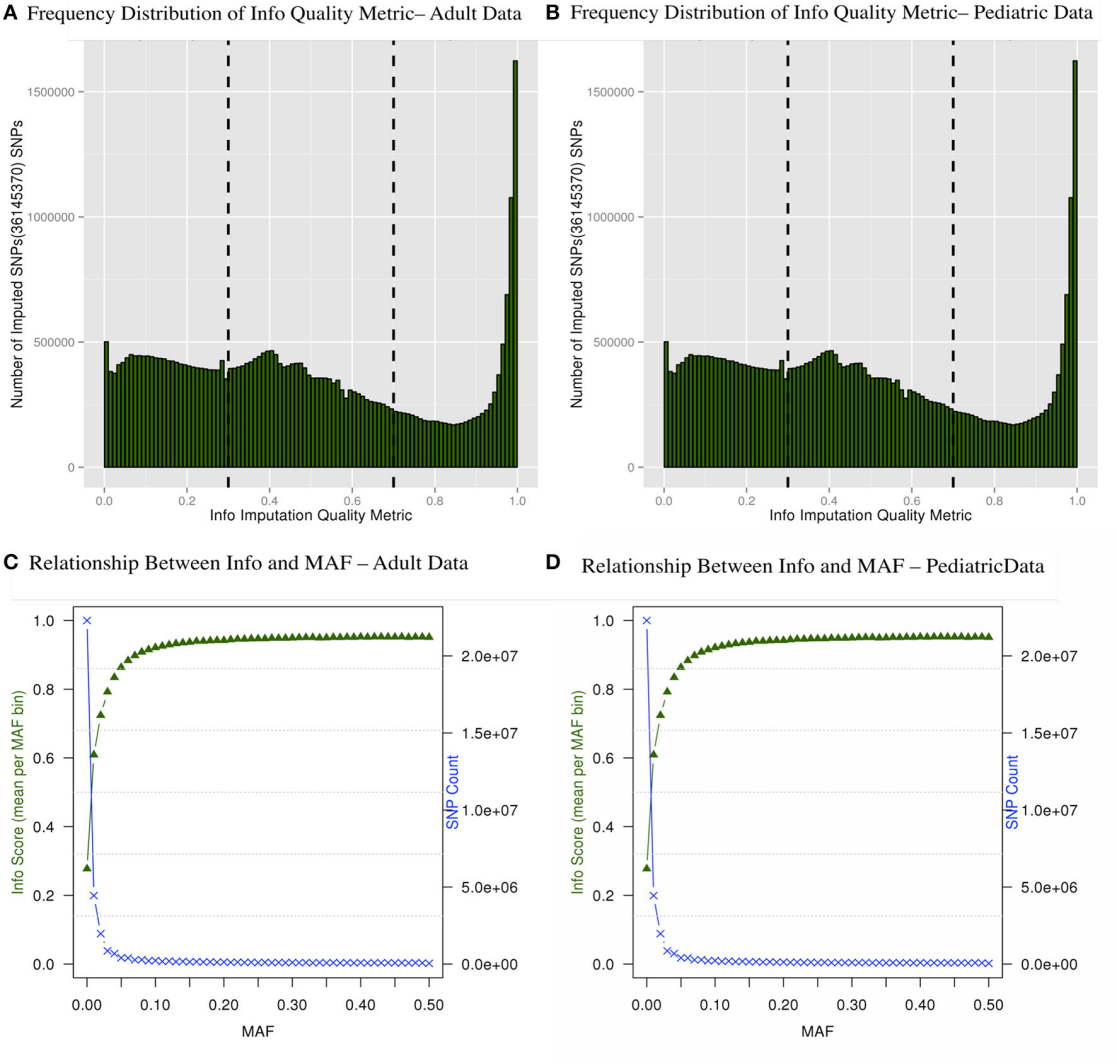
**Frequency distribution of “info” quality metric (A,B) and relationship between the “info” score and MAF are shown (C,D)**. The secondary axis indicates the count of SNPs in each MAF bin (0.01 intervals).

There are multiple results from imputation that should be evaluated before proceeding with association analyses for imputed SNPs. For instance, it is critical to consider the uncertainty of the imputed genotypes Figure 4A shows the distributions of the information (reported as “info score”) metrics for all variants in the adult imputed datasets and Figure 4B demonstrates the relationship between MAF and imputation quality for all variants in the pediatric imputed datasets by showing average “info” scores plotted in all variants grouped by MAF (bin sizes of 0.1). Although the total number of imputed variants for the adult and pediatric datasets is very similar, it was notable that there were comparatively more markers with low info score in the pediatric dataset. One potential reason for this discrepancy could be due to a much large number of genotyping platforms in the imputations of the pediatric datasets. While the average “info” scores with MAF < 0.05 fall lower than an info score of 0.8 as demonstrated in Figures 4C,D for adults and pediatric data respectively, within higher MAF bins, the average “info” scores increase to approximately 0.9. This metric demonstrates that variants imputed to have low MAF in the study samples are likely to have low MAF in the reference panel. We attempted to not include any monomorphic SNPs in the imputed dataset, our inclusion criteria was to include any imputed SNP that had at least one copy of minor allele. So the reason that we see a lot of SNPs with low “info” score is mostly due to the chosen imputation target and not any procedural error. Although there is no consensus in filtering the imputed datasets based on uncertainty of imputation, we used a variant level filter (info score >0.7) (Lin et al., 2010; Southam et al., 2011) for the downstream analyses. This is a conservative threshold, whereby we are balancing the quantity of lost data with data quality. Other studies may choose to be more liberal (info score >0.3) or even more conservative (info score >0.9).

### Quality control procedure

We performed downstream analysis of the complete, imputed merged dataset to take into account the uncertainty of the imputed genotypes. We filtered data based on info score of 0.7 after looking at the distribution of markers at all possible info scores. Because of the potential for genotyping errors in SNPs and samples with low call rates, it is essential to investigate the distribution of call rates by marker and by sample and the overlap of the two. Table 3 shows, for each marker call rate threshold, the number of SNPs dropped and the proportion of the total SNP count. Table 4 shows the sample call rate after filtering the markers with <99% call rate. At this point, we have not excluded any samples from the merged data based on sample call rates but it is very important to keep that in mind for any further analyses with these data.

**Table 3:** **Number and proportion of SNPs dropped and remaining at different genotyping call rate threshold after merged data is filtered at info score >0.7**.

**Threshold**	**SNPs dropped at threshold**	**Proportion of SNPs dropped at threshold**	**SNPs remaining at threshold**	**Proportion of SNPs remaining at threshold**
**ADULT DNA SAMPLES**				
0.95	1650764	0.1494	9400761	0.8506
0.98	3609986	0.3267	7441539	0.6733
0.99	5619475	0.5085	5432050	0.4915
**PEDIATRIC DNA SAMPLES**				
0.95	2165777	0.1275	14810393	0.8724
0.98	4983111	0.2935	11993059	0.7065
0.99	7692022	0.4531	9284148	0.5469

**Table 4:** **Number and proportion of samples dropped and remaining at different sample call rate threshold after merged data is filtered at info score >0.7 and marker call rate 99%**.

**Threshold**	**SNPs dropped at threshold**	**Proportion of dropped at threshold**	**SNPs remaining at threshold**	**Proportion of SNPs remaining at threshold**
**ADULT DNA SAMPLES**				
0.95	5	0.0001	38823	0.9999
0.98	57	0.0015	38771	0.9985
0.99	4632	0.1193	34196	0.8807
**PEDIATRIC DNA SAMPLES**				
0.95	10	0.0008	12200	0.9991
0.98	79	0.0647	12131	0.9935
0.99	497	0.0407	11713	0.9592

We have also investigated the distribution of SNPs at different MAF thresholds. We expected that imputing using the 1000 Genomes reference panel will result in a high proportion of low frequency variants. Table 5 shows the number of SNPs below and above each threshold. This summary table can be used for deciding what MAF threshold to use for association analyses. Based on power calculations, one can determine at what MAF the dataset is sufficiently powered. Subsequently, the MAF threshold can be used as a filter for analysis. We have also illustrated MAF as a filter after using a SNP call rate filter of 99%. As expected, the greater majority of the dataset consists of variants with MAF < 5%.

**Table 5:** **MAF distribution for all SNPs after applying info score (0.7) and marker call rate filter (99%)**.

**Threshold**	**SNPs dropped at threshold**	**Proportion of SNPs dropped at threshold**	**SNPs remaining at threshold**	**Proportion of SNPs remaining at threshold**
**ADULT DNA SAMPLES**				
0.05	2803753	5.1615e-01	2628296	0.4838
0.01	995223	1.8321e-01	4436826	0.8168
0.005	466779	8.5930e-02	4965270	0.9141
0.001	13979	2.5734e-03	5418070	0.997
0.0005	624	1.1487e-04	5431425	0.9998
0.0001	1	1.8409e-07	5432048	0.9999
**PEDIATRIC DNA SAMPLES**				
**Threshold**	**#SNPs dropped at threshold**	**Proportion of SNPs dropped at threshold**	**#SNPs remaining at threshold**	**Proportion of SNPs remaining at threshold**
0.05	6523370	7.3938e-01	2299322	0.2606
0.01	4631783	5.2498e-01	4190909	0.4750
0.005	3141053	3.5601e-01	5681639	0.6440
0.001	240254	2.7231e-02	8582438	0.9728
0.0005	30674	3.4767e-03	8792018	0.9965
0.0001	19	2.1535e-06	8822673	0.9999

**Figure 5:**
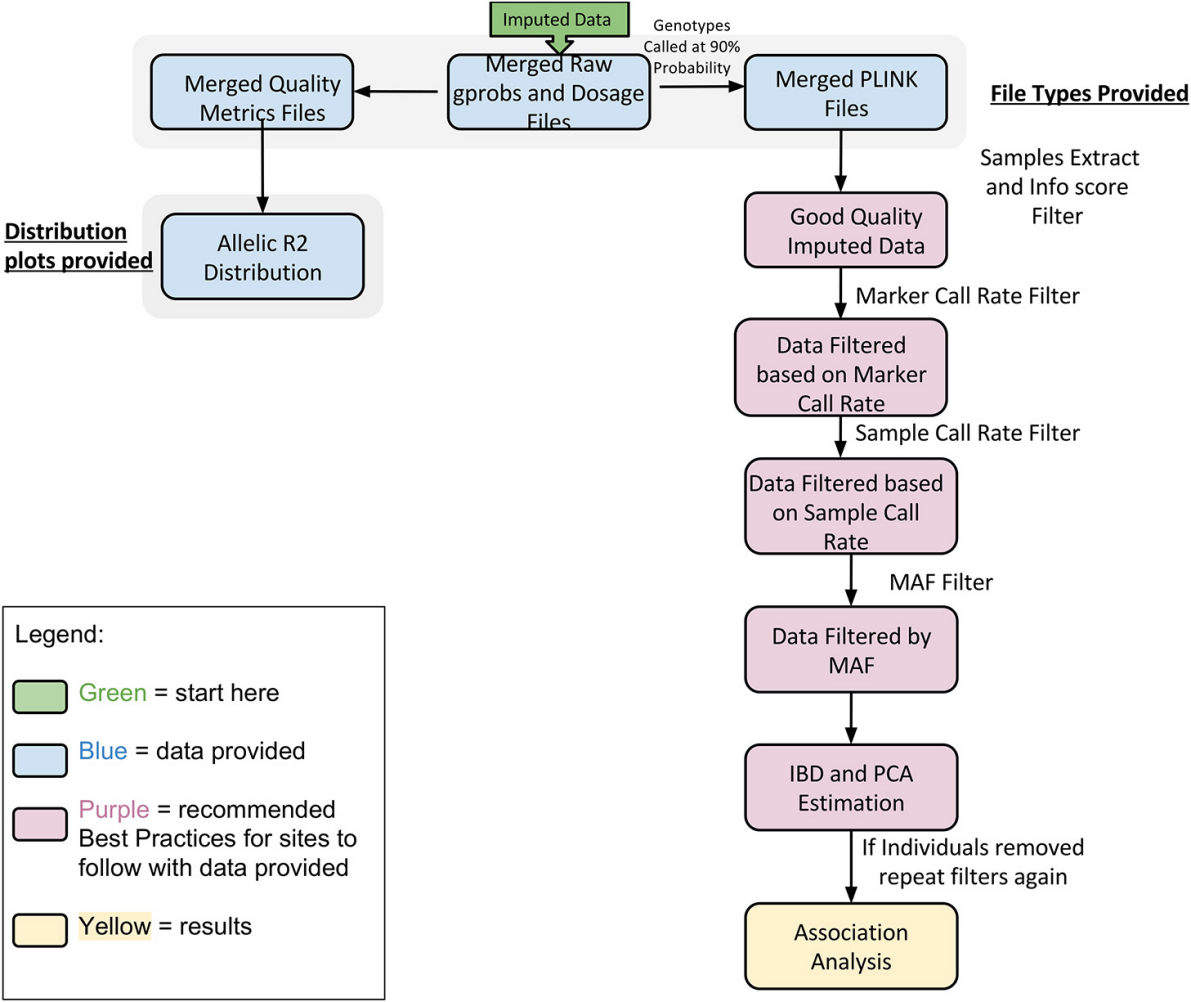
**Best practices for analyzing imputed data**.

In Figure 5 we summarize all of the “Best Practices” steps and measures for imputed data prior to using the data in any further analyses. We provided a final quality control (QC) dataset filtered at info score = 0.7 and marker call rate = 99%. We did not apply any sample call rate, and MAF filter as that depends on the type of analysis being performed. Tables 6, 7 show total counts of SNPs at each threshold we used during quality control for both the adults and pediatric datasets.

**Table 6:** **SNP summary for samples from adults participants of the eMERGE network**.

**Chromosome**	**Imputation output**	**Filter at info score 0.7**
1	2992265	857604
2	3292685	934303
3	2751021	807422
4	2725555	804138
5	2519463	725837
6	2414293	760529
7	2205621	633948
8	2174126	625724
9	1645320	479658
10	1874401	572475
11	1885432	553047
12	1818431	531244
13	1367340	414471
14	1251729	365975
15	1125278	312685
16	1204600	325272
17	1039660	276340
18	1083944	312821
19	810927	224571
20	851007	242258
21	515507	149262
22	491574	141941
Totals	38040179	11051525

“Imputation output” lists number of SNPs as result of imputation and “Filter at info score 0.7” lists number of SNPs passing info score threshold.

**Table 7:** **SNP summary for samples from pediatric participants of the eMERGE network**.

**Chromosome**	**Imputation output**	**Filter at info score 0.7**
1	2992265	1323149
2	3292686	1363591
3	2751022	1234814
4	2725555	1264290
5	2519464	1158692
6	2414294	1157434
7	2205622	990787
8	2174126	994888
9	1645320	740663
10	1874401	869202
11	1885432	851466
12	1818431	826132
13	1367340	640670
14	1251729	562490
15	1125278	491576
16	1204601	496824
17	1039661	411860
18	1083944	490988
19	810927	311239
20	851007	369387
21	515507	225258
22	491574	200770
Totals	38040186	16976170

“Imputation output” lists number of SNPs as result of imputation and “Filter at info score 0.7” lists number of SNPs passing info score threshold.

### Population structure

**Figure 6:**
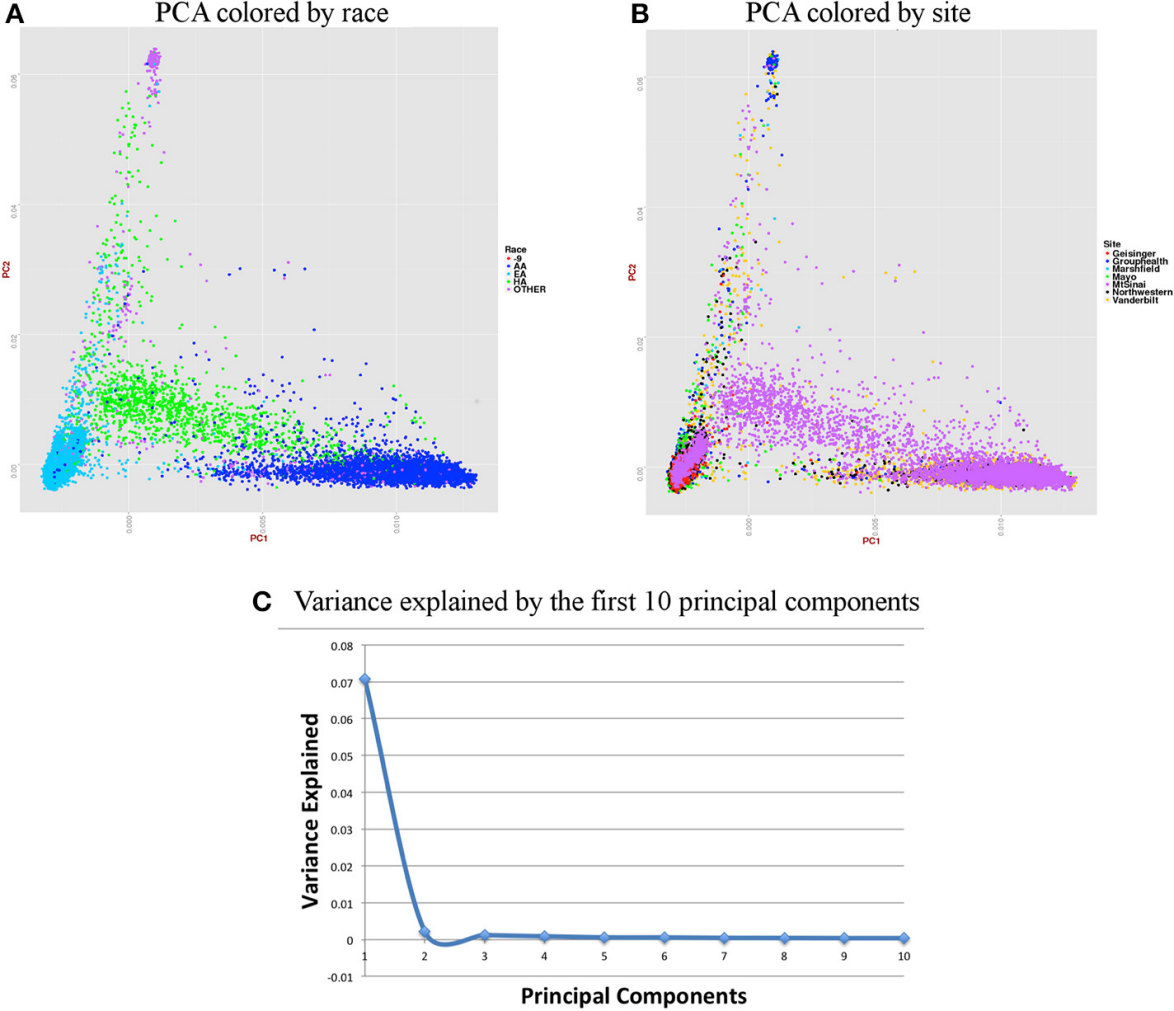
**Summary on principal component (PC) analysis for adult DNA samples. (A)** PC1 and PC2 colored by self-reported race (AA, African American; EA, European American; HA, Hispanic, Others and -9, missing), **(B)** PC1 and PC2 colored by site, **(C)** Variance explained by first 10 PCs.

**Figure 7:**
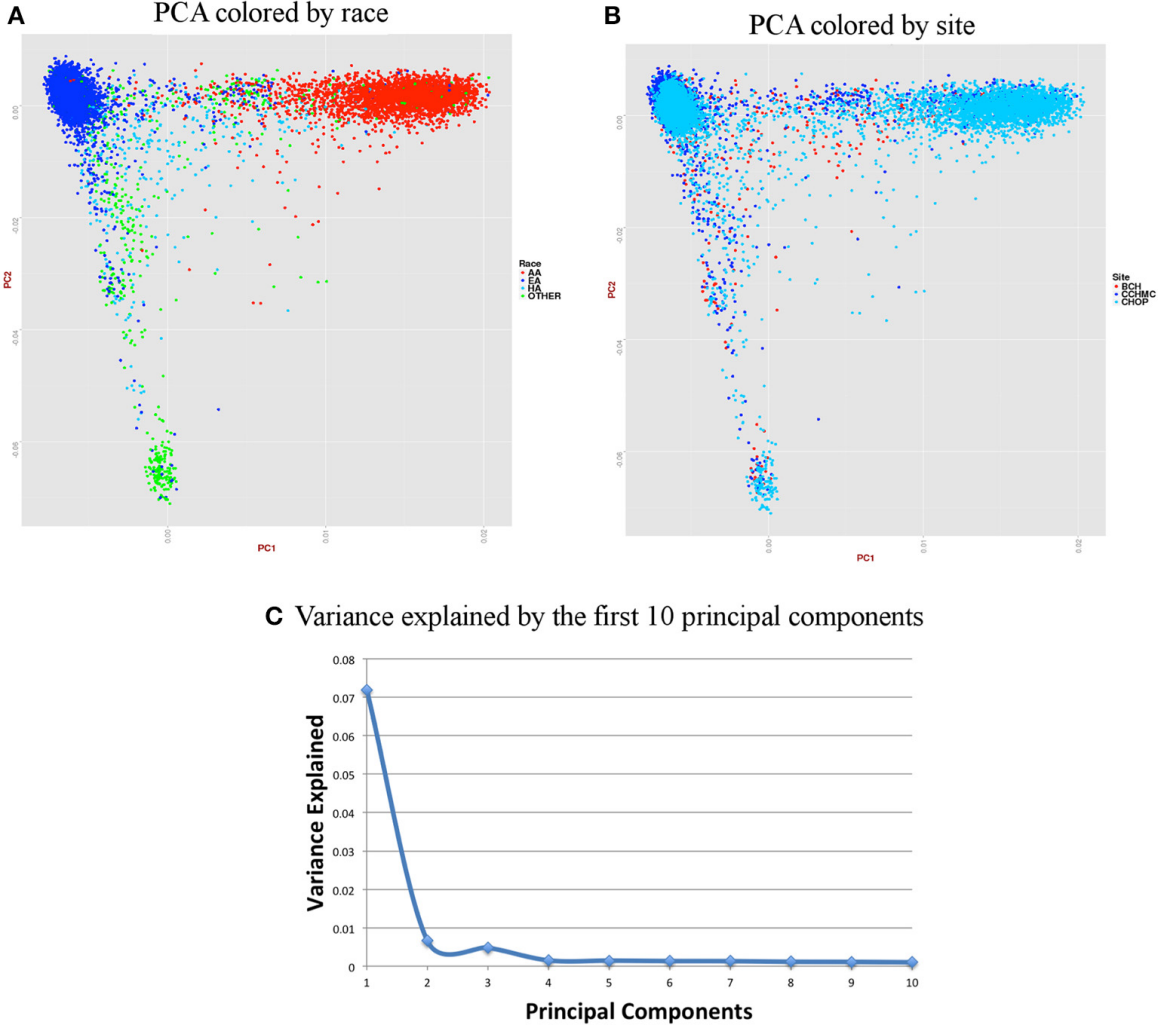
**Summary on principal component (PC) analysis for pediatric DNA samples. (A)** PC1 and PC2 colored by self-reported race (AA, African American; EA, European American; HA, Hispanic and Others), **(B)** PC1 and PC2 colored by site, **(C)** Variance explained by first 10 PCs.

For accurate imputations, it is important that the samples from imputed data cluster closely to the reference panel. We performed Principal Component Analysis (PCA) as it has been shown to reliably detect differences between populations (Novembre and Stephens, 2008). Population stratification can inflate identity-by-descent (IBD) estimates; thus, we used the KING program which is designed to circumvent the inflation of IBD estimates due to stratification (Manichaikul et al., 2010).We used a kinship coefficient threshold of 0.125 (second degree relatives) to identify clusters of close relatives, and we retained only one subject from each relative cluster. We used R package SNPRelate (Zheng et al., 2012) to carry out principal components analysis (PCA), which is a form of projection pursuit capture, because it is computationally efficient, and can be parallelized easily. Principal components (PCs) were constructed to represent axes of genetic variation across all samples in unrelated adult and pediatric datasets that were pruned using the “indep-pairwise” option in PLINK (Purcell et al., 2007) such that all SNPs within a given window size of 100 had pairwise *r*^2^ < 0.1 (for adults) and 0.4 (for pediatric) and also only included very common autosomal SNPs (MAF > 10%). We pruned data to reduce the number of markers to approximately 100,000 as previous studies have shown that 100,000 markers not in LD can detect ancestral information correctly (Price et al., 2006). These 100,000 markers included both imputed and genotyped SNPs, as the number of SNPs of overlap across the different genotyping platforms was too small to use only genotyped variants. It has been shown that PCA is most effective when the dataset includes unrelated individuals, low LD, and common variants (Zou et al., 2010; Zhang et al., 2013). We calculated up to 32 PCs, but show only the results for up to the first 10 PCs in scree plots represented in Figures 6C, 7C. It can be noted from these figures that only the first two PCs explain all of the appreciable variance and the other PCs explain very little of the variance.

For the merged imputed adult data, we removed all related individuals (IBD estimation done using KING (Manichaikul et al., 2010) kinship >0.125), performed QC, LD pruned with *r*^2^ < 0.1 and MAF > 10% to include only common variants. Thus, PCA included 37,972 samples and 1,948,089 markers. Figure 6 presents plots for PCs 1 and 2 colored by race and eMERGE site. Population structure is very well evident from these PC plots and it shows the ancestral distributions of the data from all of the eMERGE sites.

For the pediatric data, we removed all related individuals (IBD estimation done using KING^18^ kinship >0.125), performed QC, LD pruned with *r*^2^ < 0.4 and MAF > 10% to include only common variants. Thus, PCA included 11,798 samples and 162,576 markers.

Figures 6, 7 represent plots for the first two PCs colored by self-reported race or ethnicity and also represent variance explained by the first 10 PCs for both adult and pediatric datasets. Detailed PCA results on the merged eMERGE dataset are described in another publication by the eMERGE Network investigators (Crosslin et al., 2014).

## Discussion

We have performed genotype imputation to facilitate the merging of data from all eMERGE datasets. We imputed using the cosmopolitan 1000 Genomes Project reference panel and IMPUTE2 software (after a comparison with BEAGLE software). We also performed initial QC steps after merging the datasets to assess the quality and accuracy of the imputed data. Imputation results appear to be very accurate, based on the high concordance rates in the masked analysis. In addition, there was a clear distinction between the different ancestral groups, as expected, based on the PC analysis. It is very difficult to merge all of the genotype data from different platforms together prior to imputation, as a strategy to perform imputation, due to lack of sufficient overlapping markers between different genotyping platforms. Therefore, our pipeline performs imputations separately on each platform and origin of the genotype data, and then we merged the data together. We obtained very good results using this strategy and therefore consider it is an appropriate approach. It allows for the maximization of the number of genotyped markers available as study SNPs to use as the backbone to initiate imputation. It is suggested to remove all palindromic SNPs from the dataset before running any imputations to future pipelines. We performed a test on two of our datasets, running the imputation both before and after removing palindromic SNPs. Concordance check between the two runs of imputations revealed that the results were 99.8% concordant. More exploration of this issue is important for future work.

This manuscript is meant to serve as an applied, educational resource and to provide guidance for imputation. There are a number of other reviews and comparisons of different imputation packages available (Pei et al., 2008; Ellinghaus et al., 2009; Nothnagel et al., 2009; Hancock et al., 2012; Comparing BEAGLE, IMPUTE2, and Minimac Imputation Methods for Accuracy, Computation Time, and Memory Usage | Our 2 SNPs… ®). The imputed genotypes, phenotype information, accompanying marker annotation and quality metrics files for these eMERGE data will be available through the authorized access portion of the dbGaP (http://www.ncbi.nlm.nih.gov/gap). Numerous references are accessible for users wanting additional information on imputation methods, as well as recommendations for downstream analyses (Marchini et al., 2007; Servin and Stephens, 2007; Browning, 2008; Guan and Stephens, 2008; Li et al., 2009; Aulchenko et al., 2010; International HapMap 3 Consortium et al., 2010; Hancock et al., 2012; Nelson et al., 2012).

### Conflict of interest statement

The authors declare that the research was conducted in the absence of any commercial or financial relationships that could be construed as a potential conflict of interest.

## Footnotes

^1^Available at: https://www.genevastudy.org/sites/www/content/files/datacleaning/imputation/Lowe_Eur_1000G_imputation_final.pdf

^2^Available at: http://res.illumina.com/documents/products/technotes/technote_topbot.pdf

^3^Available at: https://mathgen.stats.ox.ac.uk/impute/impute_v2.html

**Table:**

CHR	SNP_ID	POSITION	allele A	allele B	Sample1_AA	Sample1_AB	Sample1_BB
22	rs149201999	16050408	T	C	0.251	0.501	0.248
22	rs146752890	16050612	C	G	0.302	0.495	0.203
22	rs139377059	16050678	C	T	0.252	0.501	0.247
22	rs188945759	16050984	C	G	1	0	0

## Abbreviations

AA: African American descent
ACT: Group Health Illumina Human Omni Express genotyped subject dataset
AffyA6: Affymetrix Genome-Wide Human SNP Array 6.0
BCH: Boston Children's Hospital, eMERGE network site
BEAGLE: BEAGLE Genetic Analysis Software Package
CCHMC: Cincinnati Children's Hospital Medical Center, eMERGE network site
CHOP: The Children's Hospital of Philadelphia, eMERGE network site
DDR3: an abbreviation for double data rate type three synchronous dynamic random access memory in computing systems
EA: European American descent
EHRs: Electronic Health Records
eMERGE: The Electronic Medical Records and Genomics (eMERGE) Network is a national consortium organized by NHGRI
GB: A unit of computer memory or data storage capacity equal to 1024 megabytes
GHz: When measuring the speed of microprocessors, a GHz represents 1 billion cycles per second
HA: Hispanic American descent
HapMap: The HapMap is a catalog of common genetic variants that occur in human beings
IBD: identical by descent (IBD)
IMPUTE2: IMPUTE version 2 (also known as IMPUTE2) is a genotype imputation and haplotype phasing program
kB: kilobyte is a multiple of the unit byte for digital information, 1024 bytes
kbp: kbp stands for kilobase pairs, a unit of length equal to 1000 base pairs in deoxyribonucleic acid or 1000 nitrogenous bases in ribonucleic acid
KING: software making use of high-throughput SNP data for determining family relationship inference and pedigree error checking and other uses
LD: linkage disequilibrium (LD) SNPs in the genome that can represent broader genomic regions
LIFTOVER: software tool that converts genome coordinates and genome annotation files between assemblies
MAF: minor allele frequency (MAF) refers to the frequency at which the least common allele occurs in a given population
MB: unit of computer memory or data storage capacity equal to 1,048,576 bytes (1024 kilobytes or 220) bytes
NHGRI: National Human Genome Research Institute
NWIGM: Northwest Institute of Genetic Medicine (NWIGM): Group Health Illumina 660W-Quad BeadChip genotyped subject dataset
PCA: Principal Component Analysis
Pos: chromosome position of a SNP
SHAPEIT2: version 2 of the haplotype inference software
UCSC: The University of California, Santa Cruz (UCSC).
